# Exposure Assessment and Biomonitoring of Workers in Magnetic Resonance Environment: An Exploratory Study

**DOI:** 10.3389/fpubh.2017.00344

**Published:** 2017-12-18

**Authors:** Anna Sannino, Stefania Romeo, Maria Rosaria Scarfì, Rita Massa, Raffaele d’Angelo, Antonella Petrillo, Vincenzo Cerciello, Roberta Fusco, Olga Zeni

**Affiliations:** ^1^Institute for Electromagnetic Sensing of the Environment (IREA), National Research Council, Naples, Italy; ^2^Department of Physics, University Federico II, Naples, Italy; ^3^Italian Workers Compensation Authority (INAIL) – Regional Technical Advisory Department Risk and Prevention Assessment (CONTARP) of Campania, Naples, Italy; ^4^Radiology Unit, Department of Support to Oncology Pathways, Diagnostic Area, Istituto Nazionale Tumori Fondazione G. Pascale (IRCCS), Naples, Italy; ^5^Department of Medical Physics, Istituto Nazionale Tumori Fondazione G. Pascale (IRCCS), Naples, Italy

**Keywords:** magnetic resonance imaging, occupational exposure, exposure assessment, human lymphocytes, micronucleus assay, mitomycin C

## Abstract

Magnetic resonance imaging (MRI) has evolved rapidly over the past few decades as one of the most flexible tools in medical research and diagnostic imaging. MRI facilities are important sources of multiple exposure to electromagnetic fields for both patients and health-care staff, due to the presence of electromagnetic fields of multiple frequency ranges, different temporal variations, and field strengths. Due to the increasing use and technological advancements of MRI systems, clearer insights into exposure assessment and a better understanding of possible harmful effects due to long-term exposures are highly needed. In the present exploratory study, exposure assessment and biomonitoring of MRI workers at the Radio-diagnostics Unit of the National Cancer Institute of Naples “Pascale Foundation” (Naples, Italy) have been carried out. In particular, exposure to the MRI static magnetic field (SMF) has been evaluated by means of personal monitoring, while an application tool has been developed to provide an estimate of motion-induced, time-varying electric fields. Measurement results have highlighted a high day-to-day and worker-to-worker variability of the exposure to the SMF, which strongly depends on the characteristics of the environment and on personal behaviors, and the developed application tool can be adopted as an easy-to-use tool for rapid and qualitative evaluation of motion-induced, time-varying electric field exposure. Regarding biomonitoring, the 24 workers of the Radio-diagnostics Unit were enrolled to evaluate both spontaneous and mitomycin C-induced chromosomal fragility in human peripheral blood lymphocytes, by means of the cytokinesis-block micronucleus assay. The study subjects were 12 MRI workers, representative of different professional categories, as the exposed group, and 12 workers with no MRI exposure history, as the reference group. The results show a high worker-to-worker variability for both field exposure assessment and biomonitoring, as well as several critical issues and practicalities to be faced with in this type of investigations. The procedures for risk assessment and biomonitoring proposed here can be used to inform future research in this field, which will require a refinement of exposure assessment methods and an enlargement of the number of subjects enrolled in the biomonitoring study to gain robust statistics and reliable results.

## Introduction

Magnetic resonance imaging (MRI), first introduced in the 1970s, has now become a common tool in modern medicine, with about 60 million MRI scans performed worldwide each year, providing critical diagnostic and anatomic information without the use of ionizing radiation (1). MRI poses a unique set of safety risk for clinical staff who receive exposures to various types of electromagnetic fields (EMFs): a static magnetic field (SMF) constantly present inside and around the MRI scanner, time-varying electric fields due to worker movements through the non-uniform SMF surrounding the scanner, radio frequency (RF) pulses in the megahertz frequency range used for signal generation, and switched gradient fields in the kilohertz frequency range that are applied for spatial encoding (2). Different clinical workers experience different exposures, with exposure patterns depending on the characteristics of the workplaces (type of scanner, layout of the MRI facility), on the job category, on the type of procedure, and patient’s state. In particular, radiology technicians, radiologists, anesthesiologists, interventionists, nurses, maintenance staff, and cleaners are exposed to the SMF and to motion-induced, time-varying electric fields, while exposure to gradient and RF pulses occurs in special cases only, e.g., during the so-called dynamic examinations, in emergencies, in attending pediatric patients, or in the case of interventional medical procedures (3). Thus, in MRI environment, health-care staff can be subjected to a multiple exposure scenario.

This assessment of exposure to EMFs in MRI environment has two aims: verifying compliance with exposure limits set by national and international regulations and providing qualitative or quantitative characterization of the exposure scenarios. The European Directive 2013/35, in its final version adopted by the Parliament and Council on June 26, 2013, has defined minimum health and safety requirements regarding the exposure of workers to EMFs at frequencies from 0 Hz up to 300 GHz (4). The Article 10 of the EU Directive identifies some derogations to the compliance with the exposure limits for several categories of workers, including those employed in the installation, testing, use, development, maintenance, or research related to MRI equipment used in clinical settings. This specific derogation is permitted, provided that the circumstances justify exceeding the exposure limit values: for example employers are required to demonstrate that MRI workers are still protected against adverse health effects and against safety risks. The exposure to motion-induced electric fields at frequencies below 1 Hz has been specifically considered in the 2014 ICNIRP guidelines (5) (with particular regard to MRI workers, but not limited to them) and is explicitly referenced in the 2015 “Non-binding guide to good practice for implementing Directive 2013/35/EU” (6).

Several different methods for the assessment of exposure to EMFs in MRI environment have been proposed in the literature. The levels of exposure to the SMF are usually measured by means of personal time recording systems (often called dosimeters), using Hall effect sensors, which are worn by the workers during the work-shift (7, 8). In some cases, personal dosimeters are also mounted with induction coils for the measurement of the temporal variation of the magnetic field (d*B*/d*t*) (9–11), although, in many cases, exposure to motion-induced electric fields is assessed by computational techniques (12–14).

Compliance with exposure limits guarantees the protection of workers against the acute effects (vertigo, stimulation of excitable tissues), however, the possibility remains that long-term exposures could result in cumulative harmful effects for health-care staff in the MRI environment, who are exposed for a few minutes to several hours per day for several years. For example, possible carcinogenic effects are undoubtedly interesting and are worthy of investigation. In this respect, studies on effects on DNA integrity are fundamental, due to the widely accepted evidence of positive correlation between significantly increased genetic damage and carcinogenesis (15).

Although, in the literature, several studies addressing the evaluation of genetic damage of SMF (16, 17), ELF (18, 19), and RF (20, 21) have been published, only a limited number of studies have been devoted to genotoxicity associated with MRI multiple EMF exposures. Such studies have been carried out mainly on cell cultures and patients or volunteers, using high SMF strengths and short exposure durations and have not led to a clear conclusion. Furthermore, the need for investigating the long-term effects at exposure levels and time duration comparable to the ones experienced by health-care staff has been highlighted (22). The same urgency for undertaking such studies has also been pointed out in the last Opinion of the EU-Scientific Committee on Emerging and Newly Identified Health Risks (SCENIHR) (23).

Cytogenetic biomonitoring, revealing chromosomal damage, is of great interest in evaluating the genotoxic effects of radiation exposure, and it has been widely carried out among the hospital workers exposed to ionizing radiation. The analysis of chromosomal damage in human peripheral blood lymphocytes (HPBLs) has been frequently used, and, according to most published studies, has revealed significant increases in medical radiation workers (24–26). For hazard identification or risk assessment purposes, the frequency of micronuclei (MN) is a reliable measure of both chromosome loss and breakage, and it has been demonstrated to be one of the most sensitive biological markers to determine the cellular response to low level of irradiation. With respect to chromosomal aberration assay, analysis of MN allows a higher number of lymphocytes to be rapidly scored (27). In addition, both MN and chromosomal aberration assays have also been employed to test the controls’ and exposed workers’ lymphocyte sensitivity to clastogenic agents such as mitomycin C (MMC) and bleomycin in different occupational environment (27, 28).

Here, we report on an exploratory study aimed to address the feasibility of procedures to characterize typical workers exposure scenarios in a MRI suite, with attention to the SMF exposure and motion-induced electric fields, and the evaluation of possible chromosomal fragility in occupationally exposed individuals. In particular, the levels of exposure to the SMF were measured by means of personal dosimeters, while exposure to motion-induced, electric fields was assessed by means of a specifically developed application tool. Moreover, the genotoxic effects and variation in cell proliferation on HPBLs from 12 MRI workers and 12 workers of the same unit with no MRI exposure history (control subjects) were evaluated by means of the cytokinesis-block MN assay. Furthermore, to test HPBLs sensitivity to a clastogenic agent, the effect of MMC treatment was also investigated.

## Materials and Methods

### Characteristics of MRI Suite

The Radio-diagnostics Unit of the National Cancer Institute of Naples “Pascale Foundation” (Naples, Italy) is equipped with a Siemens MAGNETOM Symphony, A Tim System, 1.5 T, whole body MR scanner.

Interventional or emerging procedures carried out directly under the MRI are not a common practice in the considered hospital. Therefore, only the SMF and the motion-induced electric fields have been considered for the exposure assessment.

### Exposure Assessment

#### Exposure to the SMF

The exposure of personnel to the SMF was monitored by means of calibrated, personal, wearable dosimeters (Talete, Technorad, Verona, Italy), which permit isotropic measurements of the magnetic flux density (*B*) through the use of three orthogonal Hall-effect sensors. The dosimeters are provided with a base for housing and battery charging and control software for the acquisition and transmission of measurement data. For the personal monitoring of workers enrolled in this study, the dosimeters were worn with a clip to the vest pocket, during the work-shift. The data were acquired at a sampling rate of 5 Hz and post-processed in Matlab (The MathWorks, Natick, MA, USA), and exposure results were expressed as the daily peaks of magnetic flux density (expanded maximum uncertainty, coverage factor *k* = 2, 2% of reading), by considering only the period of effective exposure to the SMF (i.e., when *B* ≠ 0). To verify compliance with exposure limits, results were compared with limit of exposure value defined by the 2013/35/EU directive for sensory effects under normal working conditions.

#### Exposure to Motion-Induced, Time-Varying Electric Fields

A numerical tool, similar to that reported by Hartwig and co-workers (14), was developed to estimate the exposure to motion-induced, time-varying electric fields of the workers enrolled in this study. The model adopted by Hartwig and co-workers considers the integral form of the Maxwell’s equation, to calculate the induced electric field as a function of the walking speed, and the current density induced in a circular loop, representing the body cross-section perpendicular to the magnetic field.

In this study, in order to obtain a more realistic representation of the human body walking in the MRI room, an elliptical, rather than circular, loop is considered, representing a section of the human body in the coronal plane. The center of the ellipsis is at the height of the central axis of the scanner. In this case, the maximum current density is given by McRobbie (1):

$$J_{\text{max}}=\frac{a^{2}b}{a^{2}+b^{2}}\sigma \frac{\text{d}B_{n}}{\text{d}t},$$

where 2*a* is the length of the major axis, 2*b* the length of minor axis of the ellipsis, and *B_n_* is the component of the magnetic induction normal to the loop surface. For a worker standing close to the bore, *a* would be in the head–foot direction and *b* is normal to this direction (1). In order to consider the trunk of a walking subject, the values of the two parameters were set to 40 cm (1) and 26.25 cm [adult, male, human model of the SEMCAD X Virtual Family (29)] for *a* and *b*, respectively. The adopted formula considers a magnetic flux density uniform over the loop surface and does not take into account the internal conductivity heterogeneity of a human body. The mean electrical conductivity (σ, in S/m) of the human tissues has been set to 0.2 S/m, a value already adopted in previous reports dealing with simplified computations at low frequencies (20).

A Matlab script was developed which reconstructs the distribution of the magnetic field on the (*x, z*) plane. Under the hypothesis that the magnetic field is generated by a magnetic dipole (30), located along the central axis of the scanner (*z* direction), the magnetic induction *B*, at this height, lays on the (*x, z*) plane and it is possible, once *B* is known, to derive the *B_x_* and *B_z_* components along the translational trajectories traveled by the loop. *B* values can be derived from the iso-gauss line map in the MRI room (as provided by the manufacturer or directly measured using a gauss meter). To confirm this, the components of B were measured (three-axis Hall Teslameter, Metrolab ETM-1) in the MRI room for two chosen translational pathways.

The assessment tool is provided with a graphic user interface, which is user-friendly for non-expert users, which can define a walking path on the magnetic field map, associate a walking speed to the movement (with a trapezoidal velocity profile), and calculate d*B*/d*t*, induced electric field, and current density.

To evaluate the exposure to motion-induced, time-varying electric fields, the workers’ activity was observed and filmed during regular working days, in order to identify the most typical pathways traveled by workers and simulate them using the application tool.

To verify compliance with exposure limits, the weighted peak (WP) approach was applied, as recommended by the 2013/35/EU Directive and by ICNIRP for non-sinusoidal signals, such as motion-induced electric fields (4, 5).

In particular, the WP index (WPI) was evaluated in the frequency domain by first computing the spectrum of the induced electric field and of d*B*/d*t* waveform and then applying the following equation:

$$\text{WPI}=\left|\sum \frac{A_{i}}{{\text{EL}}_{i}}\text{cos}(2\pi f_{i}t+\theta_{i}+\varphi_{i})\right|\leq 1,$$

where *t* is time and EL*_i_* is the exposure restriction (peak value) at the *i*th harmonic frequency *f_i_*, while *A_i_*, θ*_i_*, and φ*_i_* are the amplitude of the field, the phase angle of the field, and the phase angle of the weighting filter at *f_i_*. The weighting filter is the one indicated in Ref. (18). As concerns exposure restrictions, the basic restrictions (electric field) and reference levels (d*B*/d*t*) given in Ref. (5) were considered for frequencies below 1 Hz and those given in Ref. (18) for frequencies above 1 Hz (upon conversion of *B*_RMS_ into peak d*B*/d*t* in the case of reference levels).

### Study Subjects

The 24 workers at the Radio-diagnostics Unit of the National Cancer Institute of Naples “Pascale Foundation”, Italy, were enrolled in the study. Among them, 12 were MRI workers belonging to different professional categories (technician, health-care assistant, cleaning personnel, and medical director), and 12 had no MRI exposure history and served as reference control group.

Before blood collection, donors were asked to provide detailed information regarding, age, gender, duration of exposure, smoking habits, and family history of cancer. None of the donors were exposed to therapeutic irradiation or chemical mutagens, and all were in healthy conditions at the time of blood sampling. This study was performed in accordance with high standards of ethics and approved by the Ethical Committee of the Pascale Foundation Hospital. All individuals were informed about the aim and the experimental procedures of the study, and written consent was obtained from all participants. The main characteristics of the enrolled donors are presented in Table 1.

**Table 1:** The main characteristics of the donors enrolled in the study.

Parameter	Exposed subject	Control subject
**Number of individuals**	12	12
**Worker category**		
Technician	4	5
Health-care assistant	4	5
Cleaning personnel	2	–
Medical director	2	2
**Age (years)**		
Mean ± SD	47.25 ± 9.65	38.25 ± 13.9
Range	35–62	23–61
**Gender**		
Male %	50	58
Female %	50	42
**Smoking status**		
Non-smokers %	50	66
Smokers %	50	34
**Magnetic resonance imaging (MRI) duration of exposure (h/week)**		
Mean ± SD	11.7 ± 5.35	–
Range	5–20	
**MRI working time (years)**		
Mean ± SD	10.3 ± 5.33	
Range	3–21	
Family history of cancer	–	–

### Experimental Procedure

#### Lymphocyte Cultures

Peripheral blood samples were obtained by venipuncture from the enrolled subjects, and whole blood cultures were set up in 35 mm Petri dishes (Corning, catalog no. 430165, NY) using standard methods (31). Briefly, 0.3 ml whole blood was added to 2.7 ml culture medium consisting of RPMI 1640 medium supplemented with 15% fetal bovine serum, 100 U/ml penicillin, 100 µg/ml streptomycin, 1.0% l-glutamine, and 1.0% phytohemagglutinin for mitogenic stimulation (all materials were purchased from Biowhittaker, Verviers, Belgium). Whole blood cultures were maintained for 72 h at 37°C in a commercial incubator (model 311, Forma Scientific, Freehold, NJ, USA) in an atmosphere of 95% air and 5% CO_2_.

#### MMC Treatment

In order to identify the MMC dose to be used in the biomonitoring study, HPBL cultures from 4 out of 12 workers from the reference control group (three females and one male, aged between 23 and 31 years) were set up, and a dose–response curve was established by adding increasing MMC (Sigma, St. Louis, MO, USA) concentrations (0–200 ng/ml) at 48 h after culture initiation. MMC was dissolved in sterile physiological solution immediately before treatments and remained throughout the culture period.

#### Cytokinesis Block Micronucleus Assay

Cytochalasin B (Sigma, St. Louis, MO, USA), at a final concentration of 6 µg/ml, was added to the cultures at 44 h post culture initiation, according to standard protocols (32, 33). Cytochalasin B prevents the cells from completing cytokinesis, resulting in the formation of multinucleated cells. At the end of culture period, cells were harvested by cytocentrifugation and spun down onto slides by using a cytocentrifuge (Cytospin, Shandon, Runcorn, UK) at 1,200 rpm for 7 min, as described elsewhere (34). After fixation (80% methanol in aqueous solution for 10 min) and conventional staining with 10% Giemsa, slides were coded for a blind scoring at 1,000× magnification. In particular, for each culture, 2,000 binucleated lymphocytes with well-preserved cytoplasm were examined for the presence of MN, following the criteria suggested by Fenech (33). The results were expressed as binucleated cells (BCs) containing MN per 2,000 BCs; the number of total MN was also recorded. Moreover, on the same slides, proliferation index (PI), a measure of cell division kinetics was also calculated, as an index of cytotoxicity, by counting the percentage of cells containing 1, 2, 3, or 4 nuclei on a total of 500 cells. PI is defined as [*M*1 + 2*M*2 + 3(*M*3 + *M*4)]/*N*, where *M*1 to *M*4 represent the number of cells with one to four nuclei, respectively, and *N* is the total number of scored cells (35).

### Statistical Methods

For both MN induction and PI, comparisons among the different MMC concentrations in the range 0–200 ng/ml were carried out with one-way analysis of variance (ANOVA) for multiple comparisons at the 95% confidence level, followed by a *post hoc* Bonferroni test. Values of *P* lower than 0.05 were considered statistically significant.

The spontaneous and MMC (100 ng/ml)-induced MN incidence and PI in HPBLs from MRI-exposed workers and control workers are presented in Tables 2 and 3, respectively. For each donor, MN incidence and PI were derived by scoring 2,000 BCs and 500 total cells, respectively. Mean ± SD values are also presented.

**Table 2:** Spontaneous micronuclei (MN) incidence and proliferation index (PI) in human peripheral blood lymphocytes from magnetic resonance imaging (MRI) exposed group and control group.

MRI-exposed group				Control group			
Donor	BC with MN	Total MN	PI	Donor	BC with MN	Total MN	PI
1	17	18	1.66	1	26	29	1.82
2	10	10	1.70	2	15	16	1.87
3	19	19	1.70	3	12	13	1.57
4	17	18	1.59	4	21	21	1.86
5	11	13	1.80	5	9	9	1.60
6	8	8	1.70	6	5	5	2.10
7	12	14	1.77	7	5	5	1.61
8	9	10	1.69	8	7	8	1.46
9	8	8	1.76	9	11	11	1.60
10	10	10	1.87	10	8	8	1.89
11	8	9	1.63	11	19	20	1.76
12	5	5	1.95	12	7	7	1.80
Mean ± SD	11.17 ± 4.3	11.83 ± 4.6	1.73 ± 0.10	Mean ± SD	12.08 ± 6.8	12.67 ± 7.5	1.74 ± 0.18

*For each donor, 2,000 binucleated cells (BC) and 500 total cells were scored to derive MN incidence and PI, respectively*.

**Table 3:** Mitomycin C (100 ng/ml)-induced micronuclei (MN) and proliferation index (PI) in human peripheral blood lymphocytes from magnetic resonance imaging (MRI)-exposed group and control group.

MRI-exposed group				Control group			
Donor	BC with MN	Total MN	PI	Donor	BC with MN	Total MN	PI
1	58	65	1.47	1	35	36	1.45
2	55	62	1.48	2	43	43	1.60
3	30	31	1.52	3	29	30	1.32
4	41	43	1.41	4	44	46	1.00
5	39	42	1.58	5	32	33	1.50
6	24	24	1.42	6	28	28	1.55
7	23	23	1.53	7	27	29	1.48
8	23	26	1.53	8	21	23	1.47
9	20	20	1.49	9	28	28	1.50
10	25	27	1.68	10	39	41	1.77
11	26	27	1.51	11	54	56	1.55
12	43	43	1.60	12	35	35	1.70
Mean ± SD	33.92 ± 13.0	36.08 ± 15.1	1.52 ± 0.07	Mean ± SD	34.58 ± 9.2	35.67 ± 9.4	1.49 ± 0.19

*For each donor, 2,000 binucleated cells (BC) and 500 total cells were scored to derive MN incidence and PI, respectively*.

## Results

### Characterization of Workers Exposure

**Figure 1:**
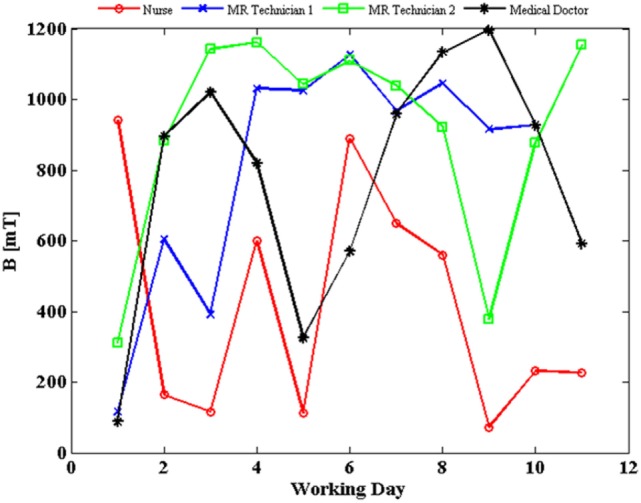
Daily peak value of magnetic flux density (*B*) experienced by four workers, measured over a period of 2 weeks (11 days of work, except for magnetic resonance imaging technician 1, blue line, for which data of 1 day were corrupted; estimated uncertainty: 2%).

The SMF measurements were carried out for all the 12 MRI workers. The results presented in Figure 1 refer to the four workers belonging to different professional categories (a medical doctor, a nurse, and two technicians) for whom we had the longest measurement period. In particular, the daily peak value of magnetic induction levels, measured over a period of 2 weeks (11 days of work), is reported for the four donors. The exposure level never exceeded the 2 T limit value defined by the 2013/35/EU directive for sensory effects under normal working conditions, although a high day-to-day and worker-to-worker variability of exposure was recorded (4). Compliance with the basic restriction given in Ref. (5) to protect against vertigo was not evaluated.

**Figure 2:**
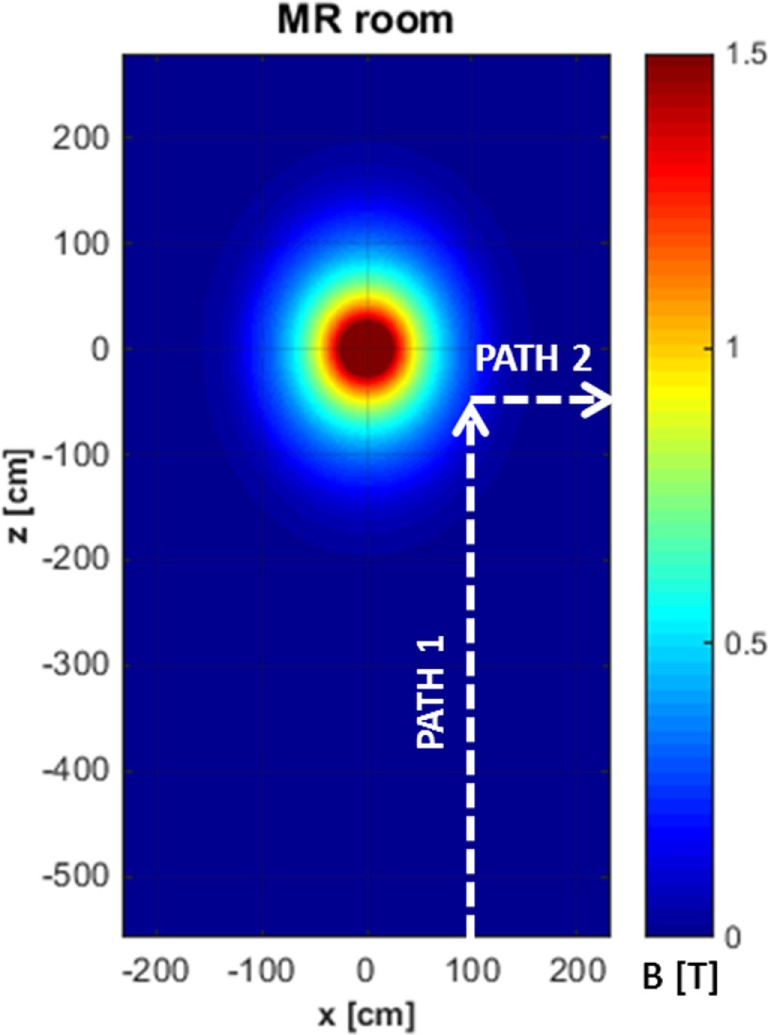
Reconstruction of the iso-gauss lines map at the height of the central axis of the scanner and representative pathways (white, dashed arrows) of workers’ movements in the magnetic resonance imaging suite.

**Figure 3:**
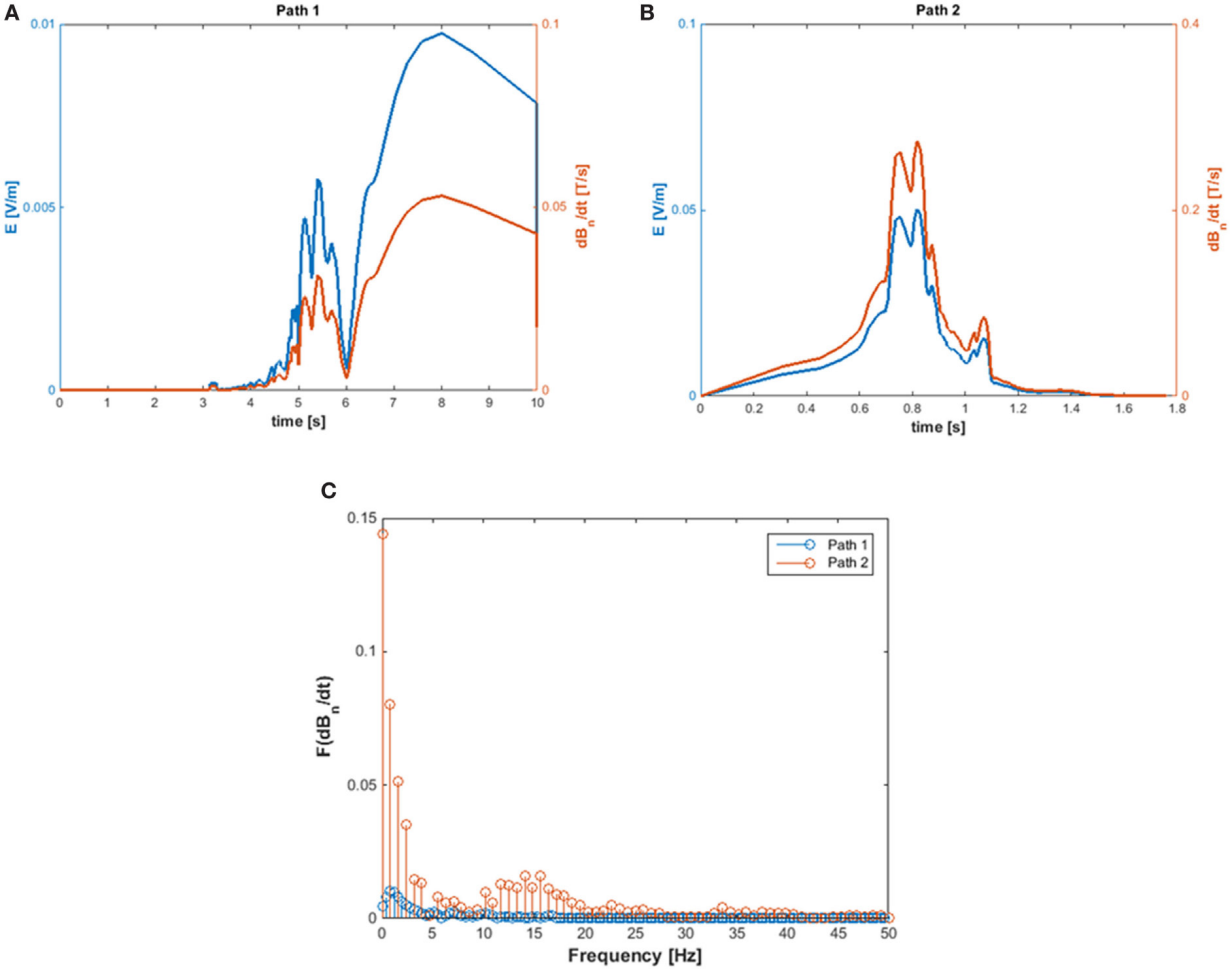
Absolute value of the induced E-field **(A)** and of d*B_n_*/d*t* **(B)** and amplitude spectra of d*B_n_*/d*T* **(C)** calculated for path 1 and path 2.

The exposure to motion-induced, time-varying electric fields was evaluated by using the developed application tool. The reconstruction of SMF distribution in the (*x, z*) plane, at the height of the central axis of the scanner, is reported in Figure 2, where the two walking paths considered for the analysis are also highlighted (white, dashed arrows). Comparison of the calculated data with measurements along the paths confirmed that at the chosen height the *B* vector lies essentially in this plane (i.e., *B* is transverse and the component along *y* direction is negligible). For both chosen walking pathways, the deviation between the transverse and the total *B* magnitude (i.e., the one derived by the iso-gauss lines) was of about 5%. In particular, path 1 starts from the entrance of the room and ends close to the scanner, representing typical movements of technicians accompanying and positioning the patients on the patient bed. Path 2, which starts close to the scanner and ends across the lateral wall of the room, can be associated with movements related to the positioning of RF coils. Along the two chosen pathways *B_n_*, i.e., the component of the magnetic induction normal to the loop surface, was supposed to be *B_z_* for path 1 and *B_x_* for path 2. It is worth mentioning that, based on this assumption, an underestimate of the total field occurs: field values for the component considered and the total field deviate by 60% close to the bore and up to approximately 20% for distances from the bore above 1 m and roughly by 15% for path 2. The walking paths were discretized with a step of 4 cm. A maximum walking speed of 160 cm/s was assumed in both cases in order to simulate quick movements of the operator and therefore considering a possible worst case. The results of simulations are reported in Figure 3, which shows the absolute values of the induced electric field (*E*), and the temporal variation of the magnetic field (d*B_n_*/d*t*) vs time, for path 1 (Figure 3A) and path 2 (Figure 3B), and the spectral components of the d*B_n_*/d*t* computed by using FFT (Figure 3C). It can be seen that the highest spectral values slightly exceed 1 Hz. In the case of the induced electric field, the computed WPI was 0.01 and 0.06 for path 1 and path 2, respectively, indicating compliance with basic restrictions. In the case of d*B*/d*t*, the computed WPI was 0.03 and 0.08 for path 1 and path 2, respectively, indicating compliance with the reference levels. The maximum induced current densities were 2.1 and 12.6 mA/m^2^, for path 1 and path 2, respectively.

### Spontaneous Genotoxicity and Cytotoxicity in the Study Subjects

The spontaneous MN incidence and the PI obtained in cells from the MRI-exposed group and the control group are reported in Table 2, which shows that the mean number of BCs containing MN and the total number of MN resulted similar in the MRI exposed workers and in control workers. A high variability in such parameters was recorded among donors: the average BC with MN was 11.17 ± 4.3 and 12.08 ± 6.8 for the MRI-exposed group and the control group, respectively. Similar results were obtained also in the case of PI with 1.73 ± 0.10 and 1.74 ± 0.18 for MRI workers and control workers, respectively.

### MMC-Induced Damage in the Study Subjects

**Figure 4:**
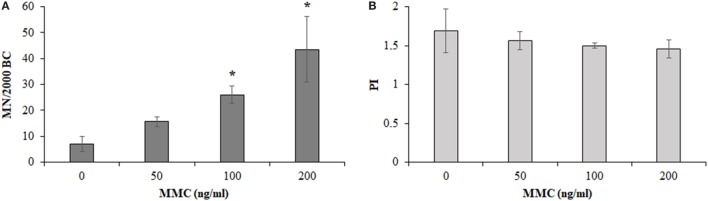
Micronuclei (MN) incidence in 2,000 binucleated cells [BCs, **(A)**] and proliferation index [PI, **(B)**] in 500 cells of control workers treated with increasing doses of mitomycin C (MMC; mean ± SD of four donors). *P < 0.05 (analysis of variance).

Figure 4 shows the results obtained from the MMC dose–response curve in HPBLs from 4 out of 12 control workers. The mean BCs containing MN, significantly increased in the MMC-treated cultures, compared to control cultures, upon increasing the MMC concentration to reach about a six-fold increase for the case of 200 ng/ml MMC concentration. Differences between untreated and the MMC-treated cultures were statistically significant at 100 and 200 ng/ml MMC concentrations (Figure 4A, *P* < 0.05, ANOVA). As expected, PI slightly decreased upon increasing the MMC dose, without reaching statistical significance, and small differences among the donors (Figure 4B) were recorded. The milder dose of 100 ng/ml MMC was chosen for the biomonitoring study to avoid the stronger damage induced by the dose of 200 ng/ml which could have hidden the hypersensitivity, if any, of the MRI workers’ HPBLs toward MMC treatment.

Table 3 presents the MMC-induced MN incidence, and PI obtained in MRI-exposed workers in comparison to non-exposed control workers. Also in this case, the mean number of BCs containing MN and the total number of MN resulted similar in the MRI-exposed workers with respect to control workers. A high variability was recorded among donors: the average BC with MN was 33.92 ± 13.0 and 34.58 ± 9.2 for the MRI-exposed group and the control group, respectively. The same holds true for PI with 1.52 ± 0.07 and 1.40 ± 0.19 for MRI workers and controls, respectively.

## Discussion

In recent years, there has been an increasing concern regarding the possible health risks arising from occupational exposure to EMFs in MRI environment, and the latest SCENHIR opinion identified the risk assessment in MRI environment as a highly important and urgent research need. Long-term prospective or retrospective cohort studies on workers that are exposed to high stray fields from the construction, operation, or maintenance of MRI devices are recommended as a high priority, in order to investigate long-term risk of disease and also to identify potential biomarkers for cancer risk and neurological diseases (23). Moreover, accurate and thorough workers’ exposure assessment is of great importance, especially in view of prospective epidemiological studies. However, exposure assessment in this environment is not a trivial task due to the highly variable, non-easily predictable exposure patterns taking place, which depend on the characteristics of the workplaces and on the personal behavior of each individual.

In this paper, possible approaches for exposure assessment and biomonitoring of workers routinely exposed to the SMF and motion-induced, time-varying electric fields of a 1.5 T MRI system have been proposed.

In particular, exposure to the SMF has been evaluated by personal monitoring of workers through wearable dosimeters. The exposure level never exceeded the 2 T limit value defined by the European Directive 2013/35 for normal working conditions (as expected for a 1.5 T MRI scanner), although a high day-to-day and worker-to-worker variability of exposure was recorded, with, on average, higher exposure levels recorded for MR technicians than for the medical doctor and the nurse, as reported also by previous work (36). Such a variability can likely be ascribed to the different activities carried out and also to different behavior between individuals. The results of SMF measurements here presented ranged between 100 and 1,200 mT and were in agreement with those reported in Ref. (36) where personal monitoring was carried out by using commercial, portable dosimeters, as well as with results in Ref. (37, 38), where SMF measurements were performed by means of a commercial and a purpose built three-axis Hall magnetometer, respectively.

The movements of workers in a spatially heterogeneous SMF can result in exposure to low frequency time-varying electric fields, inducing electrical currents within the body (39). These motion-induced, time-varying electric fields may cause transient symptoms, such as dizziness, vertigo, nausea, tinnitus, and concentration problems, which are annoying for the worker and can impair the regular working activity (40).

The direct measurement of the electric fields and currents induced in the body is not a trivial task. As a result, computational methods have been used to provide numerical estimation of the electric field/current densities and spatial distributions in the exposed body (12, 41, 42). Powerful computing resources are necessary to implement these methods, and sophisticated numerical anatomical human models, with accurate characterization of dielectric properties of different tissues, must be available to obtain reliable results. However, a simplified approach to such evaluation has been proposed by recent papers, which is based on application tools that estimate the induced electric fields and current densities once the iso-gauss line map of the MRI scanner is known and by adopting a simplified formulation of Maxwell’s equation (14, 43, 44). These methods are usually implemented with a graphical user interface (GUI), in such a way to simulate the movements of a worker on the ground plane, with assigned walking path and speed. A similar tool has been developed in this work, by using elliptical, rather than circular, loops, and by considering the transverse components of the field. Two different walking paths representing typical movements of workers in the MRI suite (derived after direct observation of the activities) have been used for assessment of exposure to motion-induced electric fields, with results compared to basic restrictions and reference levels provided by ICNIRP guidelines (with the WP approach). It is important to stress that this tool makes many assumptions, including that the body is modeled as simple loop, with homogeneous and isotropic conductivity, and that the field is generated by a single dipole. However this tool can be used to perform quick analyses, to obtain a qualitative estimate of the workers’ exposure in any MRI center, and is easily implemented by non-expert users thanks to the user-friendly GUI. For quantitative assessment, a more sophisticated model, based on accurate representations of the human body and of the source, will be necessary.

For the use of the application tool, two trajectories were considered, the first one approaching the magnet along the *z* direction and the second one leaving the magnet along the *x* direction, in such a way to mimic typical working scenarios. A trapezoidal velocity profile, with a maximum speed of 160 cm/s, was associated with both trajectories. By calculating the FFT of the d*B*/d*t*, it was observed that, for these movements, the main spectral components of d*B*/d*t* slightly exceed 1 Hz.

The results obtained in these simulations for calculated d*B*/d*t* are in agreement with those reported in previous works where d*B*/d*t* was measured by using portable dosimeters (36), commercial (37) or purpose-built (38) three-axis, Hall magnetometers.

To identify hazards for risk assessment purposes, among the markers available to monitor exposure of humans, the MN screening is a valuable tool in predicting various diseases, including cancer (45): the cytokinesis block MN assay allows for the measurement of both structural and numerical chromosomal aberrations, although further progress is needed to better understand the proper application of such a test to enable its full potential, as recently highlighted by the HUMN project consortium (46).

In the present study, both spontaneous chromosomal damage and possible hypersensitivity to the clastogenic effect of a cross-linking agent such as MMC were investigated. Twelve MRI workers were included in the study. These workers belonged to different job categories, had been working in MRI environment for various numbers of years, and were subjected to different exposure durations (11.7 ± 5.35 h/week on average), depending on job task (Table S1 in Supplementary Material). The control group comprised 12 individuals working in the same hospital unit and likely experienced the same exposure to other potential contaminants typical of the hospital environment, except for the exposure to MRI-related magnetic, electric, and electromagnetic fields. Age and gender distribution were similar among the exposed workers and the control workers. To our knowledge, this is the first time that cytogenetic biomonitoring of health-care staff who were occupationally exposed in MRI environment has been carried out. Studies reported in the literature have addressed the induction of chromosomal damage in patients who have undergone MRI examination and hence were subjected to short exposure durations. Such studies have been recently reviewed (22).

It has to be pointed out that the data reported in this exploratory study have been obtained from a small number of donors and thus do not provide the basis for any firm conclusions. Rather, the study allowed us to focus the critical issues and practicalities to be faced with in this type of investigation, which can help to inform future larger biomonitoring studies to be carried out on a suitably larger number of subjects. In particular, according to the criteria provided by Thabane et al (47), and modified for a non-clinical pilot study, the information obtained from this investigation can be grouped as follows:

(1) process—recruitment problems were found due to small number of workers employed in MRI centers and relative control groups. This can imply difficulty in satisfying very strict eligibility requirements (age, gender, and lifestyle). At the same time, no refusal in participating in the study was recorded; rather, all the invited subjects showed interest in being part of it.
(2) resources—no problems were recorded regarding the understanding of the questionnaire (results are reported in Table 1) administered to the recruited donors, and therefore, an acceptably short time was required to fill it out. The employment of the MN assay in HPBLs entails a peripheral blood withdrawal, which can easily be obtained at the same time of the periodic health surveillance controls, without an *ad hoc* blood collection, requiring only that the experimental schedule had to be matched to that of the health surveillance. Another aspect is the availability of equipment for exposure assessment. In particular, personal dosimeters to assess SMF exposure are not mandatory in MRI facilities (at least in Italy) and therefore, to cover this aspect, they were rented by the hospital. This latter aspect also translates into a not always systematic data collection. Finally, the application developed to estimate motion-induced time-varying electric fields resulted an easy-to-use tool for their rapid and qualitative evaluation. However, when additional measurements in the MRI suite were needed (e.g., to verify the data provided by the manufacturer), these were possible only when no diagnostic analysis was scheduled.
(3) scientific—the feasibility of procedures to characterize typical workers exposure scenarios in an MRI suite, with attention to the SMF exposure and to motion-induced electric fields, and to evaluate possible chromosomal fragility in occupationally exposed individuals was addressed.
(4) management—the application of the proposed procedure in several centers will allow researchers to match data coming from different sources and increase the number of observations. This in turn will also assure the reduction of variability in spontaneous and induced genetic instability.

## Ethics Statement

This study was performed in accordance with high standards of ethics and approved by the Ethical Committee of the Pascale Foundation Hospital. All individuals were informed about the aim and the experimental procedures of the study, and written consent form was obtained from all the participants.

## Author Contributions

Design of the work: OZ, MRS, AS, SR. Acquisition, analysis or interpretation of data: OZ, MS, AS, SR, RM, AP, RF, VC, and RD. Drafting or revision of the work: OZ, MS, AS, SR, RM, AP, RF, VC, and RD. Final approval of the version to be published: OZ, MS, AS, SR, RM, AP, RF, VC, and RD. Agreement to be accountable for all aspects of the work in ensuring that questions related to the accuracy or integrity of any part of the work are appropriately investigated and resolved: OZ, MS, AS, SR, RM, AP, RF, VC, and RD.

## Conflict of Interest Statement

The authors declare that the research was conducted in the absence of any commercial or financial relationships that could be construed as a potential conflict of interest.
