# Supplementary material for: Exposure Assessment and Biomonitoring of Workers in Magnetic Resonance Environment: An Exploratory Study
Source: Front Public Health. 2017 Dec 18;5:344. doi: 10.3389/fpubh.2017.00344 (PMC5741817; doi:10.3389/fpubh.2017.00344)
Supplement: Supplementary file 1 [file table_1.docx]

**Supplementary material (S1)**

| Donor |  |  | MRI employment  (Years) | Duration of exposure  (Hours/week) |
| --- | --- | --- | --- | --- |
| 1 |  |  | 21 | 10 |
| 2 |  |  | 9 | 18 |
| 3 |  |  | 14 | 20 |
| 4 |  |  | 3 | 7 |
| 5 |  |  | 7 | 7 |
| 6 |  |  | 16 | 20 |
| 7 |  |  | 16 | 12 |
| 8 |  |  | 4 | 12 |
| 9 |  |  | 9 | 12 |
| 10 |  |  | 9 | 12 |
| 11 |  |  | 8 | 5 |
| 12 |  |  | 8 | 5 |
| Mean ± SD |  |  | 10.3 ± 5.3 | 11.7 ± 5.3 |

**S1:** MRI employment (years) and weekly exposure duration (hours) of the 12 MRI workers involved in the study
